# Gross hematuria as the presentation of ureteral paraganglioma: a case report and literature review

**DOI:** 10.1186/s12894-023-01185-x

**Published:** 2023-02-17

**Authors:** Tsung-Han Cheng, Ze-Hong Lu, Che-Wei Hsu

**Affiliations:** 1 Department of Urology, National Cheng Kung University Hospital, Tainan, Taiwan; 2 Department of Pathology, National Cheng Kung University Hospital, Tainan, Taiwan

**Keywords:** Paraganglioma, Ureteral tumor, Gross hematuria, Nephroureterectomy, Bladder cuff resection

## Abstract

**Background:**

Paraganglioma of genitourinary tract is uncommon, and origin from ureter is even rarer. We aim to present a case of paraganglioma from ureter in a 48-year-old female patient, who presented with gross hematuria.

**Case presentation:**

We present a 48-year-old female who complained of gross hematuria for one week. A left ureteral tumor was found by image study. However, hypertension was unexpectedly recorded during diagnostic ureteroscopy survey. Due to persisted gross hematuria and bladder tamponade, she underwent left nephroureterectomy with bladder cuff resection. Blood pressure surged again when the tumor was surgically approached. Ureteral paraganglioma was confirmed according to pathological report. After the surgery, the patient recovered well, and no more gross hematuria was noted. She is now under regular follow-up at our outpatient clinic.

**Conclusion:**

Ureteral paraganglioma should be kept in mind not only when blood pressure fluctuates during operation, but also before we manipulate the ureteral tumor when gross hematuria is the only sign. Whenever the presumption of paraganglioma is raised, laboratory evaluation and anatomical or even functional imaging should be considered. The concomitant anesthesia consultation before the surgery should not be deferred, either.

## Background

Paraganglioma of urinary tract is rare, and it is reported to mostly originate from bladder, kidney and prostate [1]. Among these cases, the ureteral paraganglioma is even rarer, and only 6 cases have been reported [1–6]. When paraganglioma is suspected, the laboratory work-up and imaging study will be considered to rule out this disease. However, there are still some clinical dilemmas that may mask this crucial disease. In such situation, we may ignore the diagnosis and encounter difficulties in treatment. Herein, we present a case of ureteral paraganglioma in a 48-year-old female patient, who complained of severe gross hematuria for one week.

## Case presentation

A 48-year-old female, who denied any systemic diseases, presented with severe gross hematuria and voiding difficulty for one week and came to the emergent department of our hospital for help.

Tracing back her history, she had visited regional hospital for gross hematuria several weeks ago before she came to us. Urinary bladder tamponade was noted and manual bladder irrigation with blood clots evacuation was also performed. Further imaging with contrast-enhanced computed tomography (CT) scan showed left hydronephrosis, and a round, well-defined retroperitoneum mass was noted adhering to left upper ureter, lying at left para-aortic region and the aortic bifurcation level. It was measured about 4.3 cm, and displaced the ureter medially, and the ovarian vein anteriorly. It also indented on the psoas muscle. The mass showed homogenously vivid after contrast enhanced. Massive blood clots were found within renal pelvis and bladder. Diagnostic semi-rigid ureteroscopy was arranged, and serpentine blood clots were found within left ureter. However, the ureteroscopy could not pass the compression site, where the sharp angulation existed. Unexpectedly, blood pressure surged when they tried to pass the stricture site with ureteroscope. The diagnostic ureteroscopy was incomplete and double J stent was placed for hydronephrosis. She was then referred to our hospital for further management.

At our emergency department, fever up to 39.2’c was recorded, but the blood pressure was within normal range (112/71 mmHg). The accompanied symptoms include dysuria, urinary frequency, and urgency. Basic hemogram revealed leukocytosis and elevated CRP level, but there was no deterioration of renal function. Meanwhile, pyuria was also recorded through urine analysis.

**Fig. 1:**
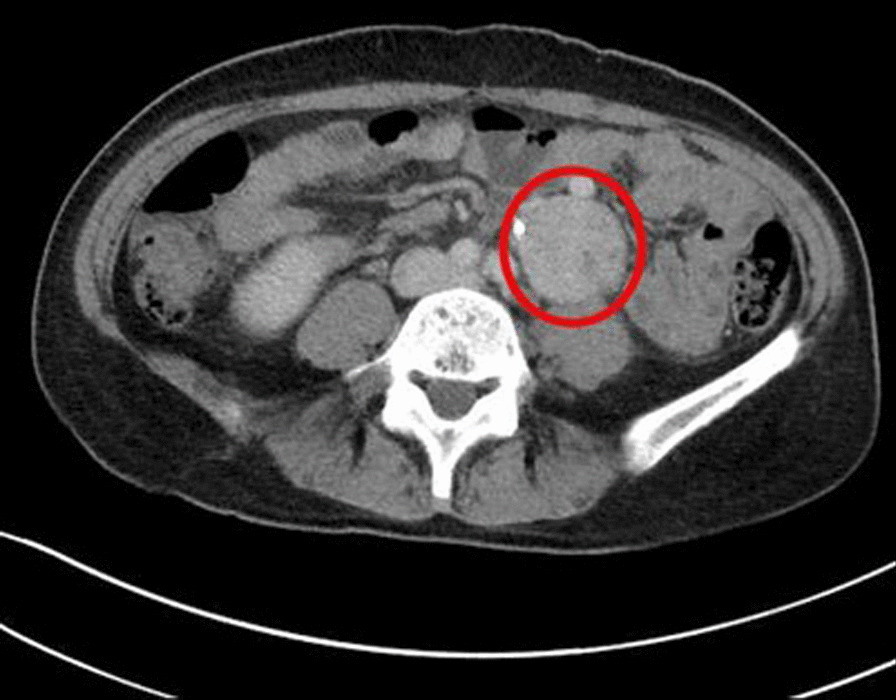
A round shape, up to 4.3 cm heterogenous enhanced mass over left retroperitoneum next to left ureter which is stented by double J tube in transverse view of contrast abdominal CT

Reviewing her CT at regional hospital, we could see left hydronephrosis with a left retroperitoneal tumor about 4.3 cm adhering to and compressing left upper ureter with massive blood clots in bladder (Fig. 1). We also noticed that blood pressure surge during the ureteroscopy, so the presumption of paraganglioma was raised. Therefore, we tried to collect urine vanillylmandelic acid (VMA) and catecholamine for metabolic workup. Nevertheless, it was due to severe hematuria and bladder tamponade that we could not complete this study.

Fever gradually subsided after adequate antibiotics treatment. However, gross hematuria persisted with intermittent Foley catheter obstructed by blood clots. Manual bladder irrigation with blood clots evacuation was performed several times but in vain. Accordingly, her hemoglobin level dropped from 11 to 8 g/dL, so adequate blood transfusion was also given. During the clinical course before further surgical intervention, her blood pressure was kept within normal range.

After discussing with the patient, emergent excision of left retroperitoneal mass, which compressed and adhered to upper ureter, and possible nephroureterectomy with bladder cuff excision was arranged.

Under the impression of paraganglioma, though laboratory workup failed to be completed, we informed this situation to the anesthesiologist and made sure blood pressure was under cautious monitoring. During the operation, a round tumor was found adhesive onto left upper ureter, and severe adhesion between the tumor and gonadal vein was also identified. Extremely high blood pressure (> 200 mmHg) was detected during tumor manipulation even after multiple antihypertensive agents given by anesthesiologists. After tumor blood supply ligation, blood pressure restored to normal range rapidly. The patient recovered smoothly after the operation, and no hypertension was recorded after the surgery. The hemoglobin level also came back to normal range. Finally, she was discharged 9 days after the operation.

**Fig. 2:**
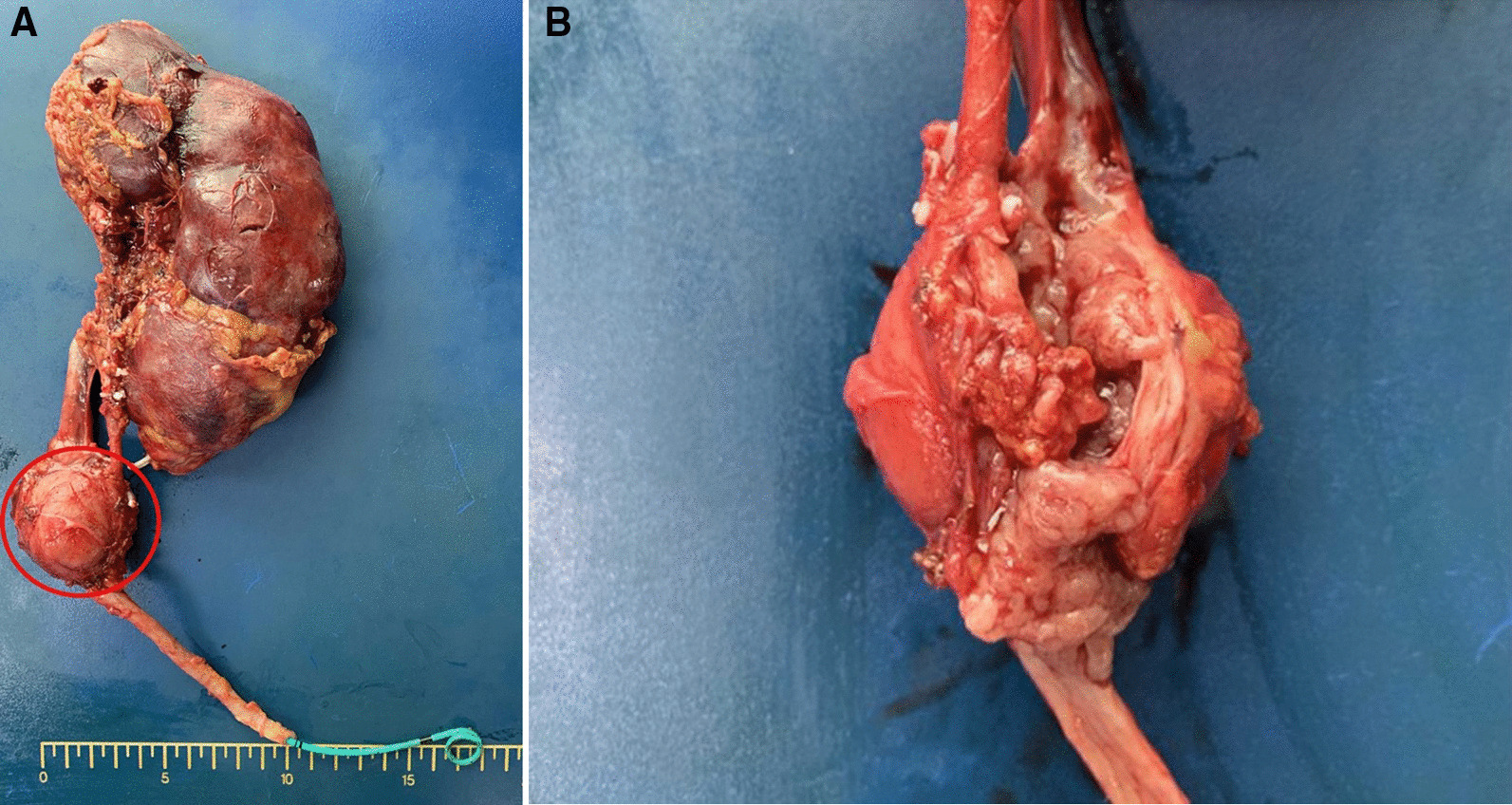
**A** The gross photo of specimen revealed round shape, elastic consistency mass attached left upper ureter and left gonadal vein. **B** The gross appearance of paraganglioma cut section

**Fig. 3:**
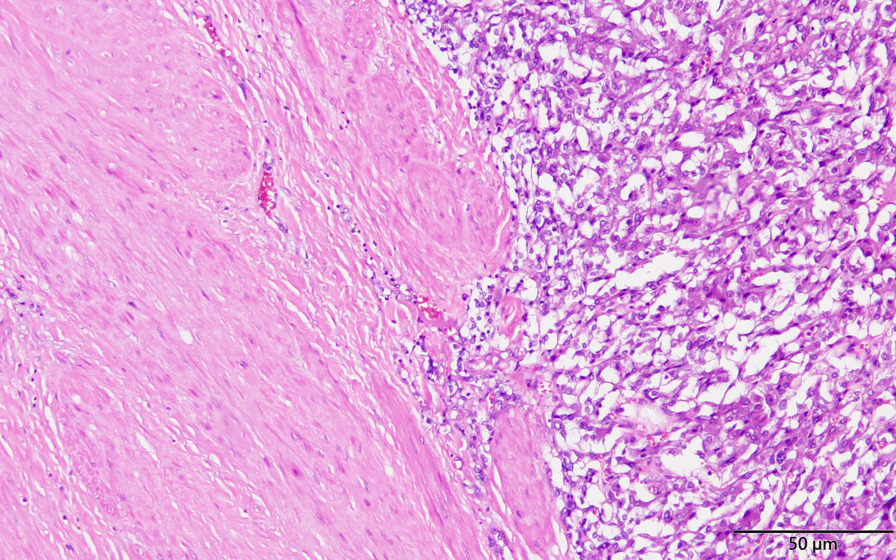
Microscopic finding of the tumor reveals tumor cells originating from outer muscle layer and ureteral adventitia

For the surgical finding, we found the specimen was a 4 × 3.5 × 3 cm elastic mass, which attached to left ureter, compatible with CT findings (Fig. 2). Microscopically, the tumor cells originated from outer muscle layer and adventitia of left ureter (Fig. 3). The neoplastic cells are round to polygonal with abundant eosinophilic granular to clear cytoplasm. The tumor cells are positive for Chromogranin A and Synaptophysin.

Six months after the surgery, the level of 24 h-urine catecholamines were within normal range. The follow-up computed tomography also revealed no recurrence of retroperitoneal tumor.

## Discussion

Paraganglioma, which is also described as pheochromocytoma by previous articles of bladder, has been first described in 1953 by Zimmerman [7], and it stands for a very rare subtype of bladder tumor. It represents only 0.06% of bladder tumor and 1% of all pheochromocytoma [8]. The onset age is around 20–40 years old, and is more prevalent in women than in men [9]. On top of that, paraganglioma of ureteric origin is rarer, and only six cases around the world have been reported [1–6]. These cases are listed in Table 1.

**Table 1:** Literature review of ureteric paraganglioma

Author	Age	Gender	Tumor site	Tumor size	Catecholamine	Preoperative hypertension	Presentation	Management
Samuel et al. (1971)*	12	Female	Right ureter	4 × 3 cm	N/A	Yes	Severe hypertension with ureteropelvic junction obstruction	Tumor excision with nephroureterectomy
Victor et al. (1973)*	62	Male	Right ureter (ureterocele)	5.5 × 3.5 cm	N/A	Yes	Palpitation, headache and hypertension	Tumor excision with ureteroneocystostomy
Kumar et al. (2009)	12	Male	Right ureter	4 × 3 cm	Urine VMA: normal	N/A	Gross hematuria	Tumor excision and ureteroneocystostomy
Namrata et al. (2011)	33	Male	Left ureter	6 × 5 × 4 cm	Urine VMA: increased	Yes	Palpitation with hypertension	Tumor excision with parts of ureter
Lakshmi et al. (2017)	40	Female	Left ureter	1.5 cm	N/A	No	Flank pain with hydronephrosis	Tumor excision with parts of ureter
Zairan et al. (2020)	46	Female	Left ureter	3.9 × 2.6 cm	N/A	Yes	Hydronephrosis	Tumor excision with parts of ureter

*N/A* non-available; *VMA* vanillylmandelic acid

*These articles use pheochromocytoma instead of paraganglioma

The previous reports showed that the ureteral paraganglioma could be measured from 3.5 to 6 cm and could be located from renal hilum to the site of insertion to bladder.

The postoperative follow-up time of all six cases was around 1–2 years, and no tumor recurrence was found according to these studies.

For paraganglioma arising from genitourinary tract, bladder is the most common site of growth. The malignancy rate of these tumors has been described as 15–20%.

Among these cases of GU tract paraganglioma, they could be adequately managed by surgical excision [1].

The majority of initial presentation is hypertension or related symptoms and can be followed by hydronephrosis. Gross hematuria as initial symptom is relatively rare and differential diagnosis remains a challenge in clinical practice. The uncommon presentation in our case could be explained through the view of histopathology, by which the origin of the tumor from muscle layer of ureter might explain this phenomenon.

Since hypertension is the most common symptom in previous reports, it is crucial to raise such a presumption whenever fluctuating blood pressure is recorded when evaluating the disease, especially during manipulation of the tumor. We’ve learned a lesson from our case that although normo-tensive status was detected in most times in the clinical course, blood pressure surged during diagnostic ureteroscopy stood for a critical hint, through which ureteral paraganglioma should be considered.

From previous studies, we realized that plasma metanephrines are the most accurate parameter for detection of pheochromocytoma, whose sensitivity can reach 99%, and urinary fractionated metanephrines, with 97% sensitivity, are the most accurate parameters for detection of pheochromocytoma, and they perform better than urinary VMA from our case, whose sensitivity is only 77% [10]. The choice of urinary VMA instead of plasma metanephrines or urinary metanephrines in our case came from the limit of practice in our clinical laboratory section. However, we believe that plasma metanephrines or urine metanephrines are the first choice of assessing possible pheochromocytoma if it is available clinically. In our case, we did try to make more specific diagnosis of ureteral paraganglioma, but urgent surgical intervention was indicated owing to persisted hematuria and refractory blood clots evacuation at bedside, let alone her hemoglobin level dropped quickly. In this case, the clinical course and surgical indication was and should be explained comprehensively to the patient, since the operation for ureteral paraganglioma is more risky than usual NU.

**Fig. 4:**
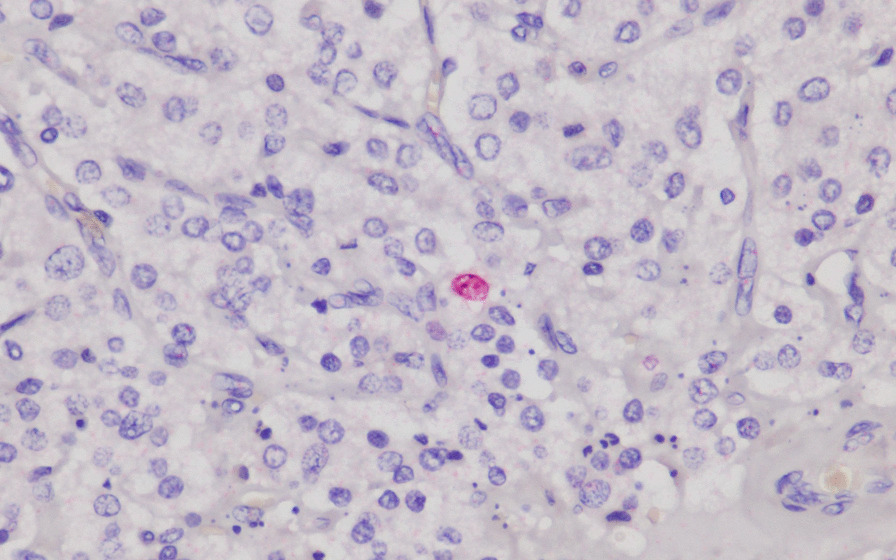
Immunohistochemical study of Ki-67. The figure shows low positive rate of Ki-67 stain

**Fig. 5:**
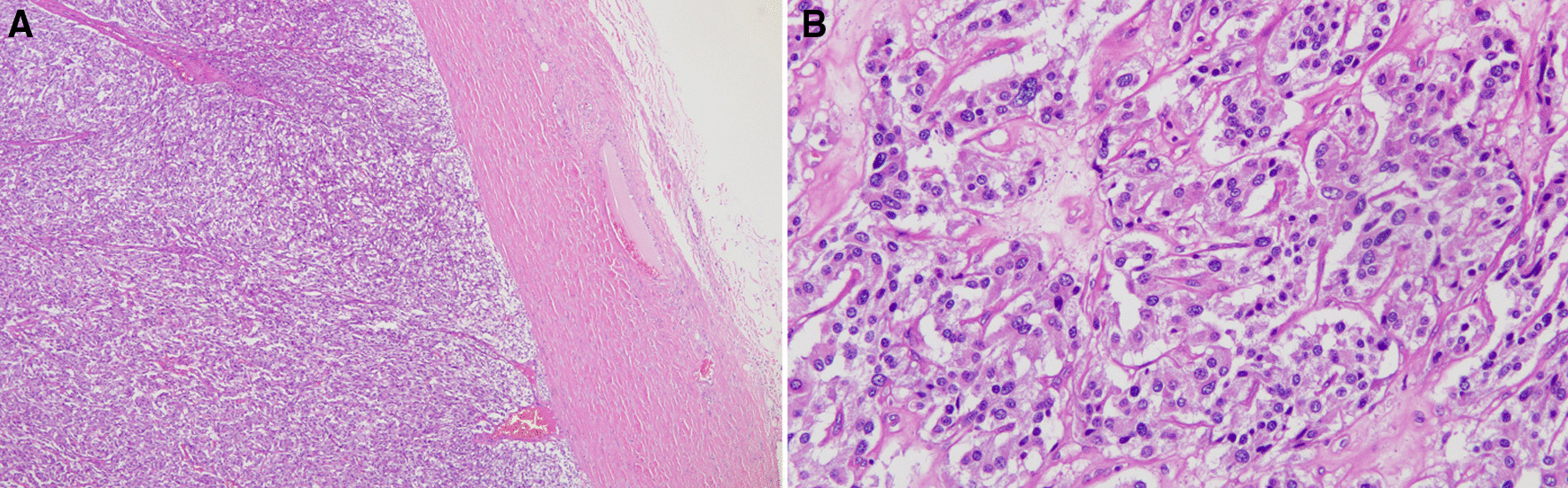
**A** Scanner view shows no capsular invasion or adipose tissue extension. **B** No large nest is seen through high-power field. Areas of small nests (“zellballen”) could be observed as features of pheochromocytoma

It is well known that pathological evaluation for malignant pheochromocytoma or paraganglioma is difficult. The Ki-67 stain, and Pheochromocytoma of the Adrenal gland Scaled Score (PASS) score is what we can facilitate in order to offer more accurate follow-up [11]. Grossly, we identified an ovoid, firm and well-circumscribed tumor through the specimen. Its immunohistochemical panel of Ki-67 is less than 1% (Fig. 4). Ki-67 is known to be quite variable in both benign and malignant pheochromocytomas, through which both groups can express less than 1% historeactivity [11]. In addition, histologic features from PASS score also offer a number of clues for us to separate the aggressiveness of the tumor. Whether it is benign or malignant, small nests (“zellbellen”) can be observed in all pheochromocytomas. In our case, the tumor was comprised of large nests, and there was no central or confluent tumor necrosis. High cellularity didn’t exist, and no cellular monotony was identified. On top of that, tumor cell spindling was not observed, and mitotic figures was less than 3/10 high-power fields. There was no extension of tumor into adipose tissue. Neither vascular nor capsular invasion was found. At last, no profound nuclear pleomorphism or nuclear hyperchromasia was noted (Fig. 5). Through the above histologic features, the PASS score indicated a benign fashion (PASS < 4) [11]. Furthermore, the cytokeratin stain was negative, which exclude the origin from epithelial cell of ureter.

After the operation, image follow-up at clinic is important for us to evaluate any signs of recurrence or metastases. We can learn from former studies that the survey and follow-up of paraganglioma include anatomical and functional imaging. The former includes CT and magnetic resonance image (MRI), which could reveal the contour and content with high sensitivity. The latter is composed of [^123^I]-metaiodobenzylguanidine single photon emission computed tomography (^123^I-MIBG SPECT), which provides high specificity for paraganglioma identification, and positron emission tomography (PET) with radiopharmaceuticals such as 6-[^18^F]-fluorodopamine (^18^F-FDA) and [^18^F]-fluoro-dihydroxyphenylalanine (^18^F-FDOPA). They have also been used to locate paraganglioma and can also be seen as a routine functional imaging of paraganglioma. What’s more, comparing to ^123^I-MIBG SPECT, [18F]-fluoro-2-deoxy-D-glucose (^18^F-FDG) PET seemed to detect metastases better with high specificity. The uptake of ^18^F-FDG could indicate hereditary syndrome that underlies the paraganglioma [12].

More recently, the European Association of Nuclear Medicine recommended functional imaging for paraganglioma with ligands like ^18^F-fluorodopamine, ^18^F-dihydroxy-phenalalinine (DOPA), ^18^F-FDG or ^68^Ga-DOTATOC/DOTATATE/DOTANOC. The 18F-FDG stands out with a sensitivity of 80–100%, but with less specificity to detect extra-adrenal disease. Ga-DOTATOC and ^18^F-DOPA have been shown to be more accurate than ^123^I-MIBG, especially in small lesions and in extra-adrenal diseases [13, 14].

Preoperative diagnosis of paraganglioma may sometimes fall into dilemma if clinical symptoms are occult and laboratory study is limited. In our intraoperative hypertension is the only hint to indicate paraganglioma, and we also failed to gain the result of urinary VMA due to severe hematuria and we also couldn’t check urine metanephrines due to the limitation of facility. Other prompt and clinically feasible survey of paraganglioma may be needed in the future if urgent surgical intervention is inevitable.

## Conclusion

Clinical presentation of ureteral tumor with gross hematuria raises the suspicion of urothelial carcinoma other than paraganglioma. However, the differential diagnosis of paraganglioma should be kept in mind if blood pressure surged when manipulating around the main tumor during ureteroscopy or even surgical manipulation. Even if gross hematuria is the only sign at first, this diagnosis of paraganglioma shouldn’t be omitted. Besides, anatomical or even functional study should always be done before invasive procedure if it is clinical available. Careful anesthesia consultation should not be omitted before the surgery. After the operation, the follow-up image study, renal function and urine test should also be monitored.

## Data Availability

All data generated or analysed during this study are included in this published article.

## Abbreviations

NU: Nephroureterectomy
CT: Computed tomography
CRP: C-reactive protein
VMA: Vanillylmandelic acid
PASS: Pheochromocytoma of the Adrenal gland Scaled Score
MRI: Magnetic resonance image
MIBG: Metaiodobenzylguanidine
SPECT: Single photon emission computed tomography
PET: Positron emission tomography
FDA: Fluorodopamine
FDOPA: Fluoro-dihydroxyphenylalanine
DOPA: Dihydroxy-phenalalinine
FDG: Fluoro-2-deoxy-D-glucose
